# Uncovering complex correlations between multidimensional factors and breastfeeding duration using XGBoost

**DOI:** 10.1371/journal.pone.0354857

**Published:** 2026-08-07

**Authors:** Lijing Yang, Wanju Wang, Yajuan Chen

**Affiliations:** Department of Obstetrics and Gynecology, Wuhan Children’s Hospital (Wuhan Maternal and Child Healthcare Hospital), Tongji Medical College, Huazhong University of Science & Technology, Wuhan, Hubei, China; Korea University - Seoul Campus: Korea University, KOREA, REPUBLIC OF

## Abstract

**Background:**

Breastfeeding duration is influenced by a complex interplay of obstetric, physiological, and sociodemographic factors. Traditional linear statistical models often struggle to capture the high-dimensional, non-linear synergistic effects among these variables, while complex deep learning models are prone to overfitting on small clinical datasets.

**Methods:**

To address these challenges, this study proposes an interpretable machine learning framework based on **eXtreme Gradient Boosting (XGBoost)**. We analyzed clinical follow-up data from 210 postpartum women. The framework incorporates **Explicit Polynomial Feature Engineering** to construct high-order interaction terms and utilizes **SMOTE** to resolve class imbalance. Crucially, **SHAP (SHapley Additive exPlanations)** analysis was employed to provide bidirectional feature attribution. **Results:** The proposed model achieved robust, cross-validated performance with an **Accuracy of 76.2% (95% CI: 64.3%−88.1%)**, an **AUC of 0.72 (95% CI: 0.54–0.89)**, and a high **Recall of 0.92** for the long-term breastfeeding group (≥ 11 months), outperforming traditional baselines. SHAP analysis identified critical non-linear risk patterns. Notably, the interaction between **“Delivery Mode** × **Milk Stasis Duration”** demonstrated that the protective effect of vaginal delivery mitigates stasis risks, whereas Cesarean sections disproportionately amplify the inhibitory effect of milk stasis. Additionally, the negative interaction of **“Education** × **Feeding Type”** highlighted the compounded risk for highly educated mothers transitioning to mixed feeding.

**Conclusions:**

This study confirms that an interpretable XGBoost framework with explicit interaction features effectively overcomes the modeling bottlenecks of small-sample clinical data. By uncovering hidden synergistic risk factors, it provides a data-driven basis for precision lactation support, advocating for targeted interventions for specific high-risk profiles rather than generic education.

## Introduction

Breastfeeding is globally recognized as the “gold standard” for infant nutrition, playing an irreplaceable role in building the infant immune system, promoting neurocognitive development, and reducing long-term disease risks for both mothers and infants [1,2]. Although the World Health Organization (WHO) and various national health departments strongly recommend exclusive breastfeeding for the first six months of life, global adherence remains suboptimal [3,4]. In China, the rate of exclusive breastfeeding often drops precipitously within three months postpartum due to multidimensional barriers such as return-to-work pressure, insufficient social support, and physiological health issues [5–7]. Therefore, exploring the key risk factors leading to early weaning is of urgent significance.

Breastfeeding is a complex behavior regulated by multidimensional factors. Previous epidemiological studies indicate that sociodemographic characteristics [8,9], obstetric factors [10,11], and perinatal physiological indicators are fundamental variables. Among them, milk stasis and mastitis are widely considered the main physiological barriers [12,13]. However, traditional statistical studies often treat these factors as independent variables, employing linear models for analysis [14]. Recent studies point out that there are complex synergistic effects among risk factors which are difficult for traditional linear models to capture [15,16].

With the rise of medical big data, Machine Learning (ML) has become an important tool for clinical predictive modeling [17]. Multiple studies confirm that algorithms such as Random Forest and Support Vector Machines (SVM) significantly outperform traditional regression models in predicting breastfeeding outcomes [15,16]. Specifically, XGBoost provides a highly scalable and robust tree-boosting system capable of handling complex tabular data efficiently [18].

Despite the immense potential of machine learning, its practical clinical application faces two major challenges: the dilemma of small sample sizes where complex models are prone to overfitting [19], and the “black-box” problem lacking interpretability [20,21].

This study aims to fill this gap by proposing an interpretable XGBoost interaction prediction framework tailored for small-sample clinical data. We explicitly construct high-order interaction terms using polynomial feature engineering, address the class imbalance problem via SMOTE [22], and introduce bidirectional feature attribution analysis. This study is dedicated to revealing the implicit interaction mechanisms between physiological and social factors, providing transparent and actionable intervention targets.

## Materials and methods

### Study design and data collection

Data were accessed for research purposes from 20/11/2025 to 25/12/2025. This retrospective cohort study utilized clinical follow-up data collected from May 15, 2018, to August 15, 2024. The study population consisted of 210 postpartum women who had completed breastfeeding or had been breastfeeding for at least 11 months at the time of the survey. The primary outcome variable was the duration of breastfeeding, defined as a binary classification target based on a clinically significant threshold of 11 months (*Target* = 1 for duration ≥ 11 months; *Target* = 0 for duration < 11 months). While global guidelines recommend exclusive breastfeeding up to 6 months and continued breastfeeding up to 12 months, our choice of 11 months reflects the local epidemiological context in China, where a significant drop-off in breastfeeding occurs as maternity leave concludes and mothers return to the workforce. This threshold allows the model to accurately capture the predictive signatures of mothers who successfully navigate these socioeconomic barriers.

Data collection encompassed a multidimensional set of predictors, categorized into three domains: Sociodemographic factors, Obstetric and physiological history, and Lactation-specific metrics. To ensure data quality, participants with incomplete medical records were excluded.

**Ethics Statement:** The study was conducted in accordance with the Declaration of Helsinki, and approved by the Ethics Committee of Wuhan Children’s Hospital (protocol code 2025R147-E01 and date of approval 17 November 2025). Patient consent was waived due to the retrospective nature of this study, as the research involved the review of existing medical records and presented minimal risk to the subjects.

### Explicit feature interaction engineering

We utilized the Yeo-Johnson power transformation to stabilize the variance of continuous variables. Subsequently, we explicitly constructed second-order interaction terms (Degree = 2) for all input variables using the polynomial expansion technique. This process expanded the original feature space to include combination features (e.g., *Age* × *Milk Stasis Frequency*). To prevent the “curse of dimensionality,” a Random Forest classifier was employed to evaluate the contribution of each generated interaction term. Only the top-ranked features were retained for the final modeling phase by utilizing the median importance score as a rigorous cutoff threshold to reduce noise.

### Addressing data imbalance with SMOTE

Clinical datasets frequently exhibit class imbalance. To mitigate this bias, we employed the **Synthetic Minority Over-sampling Technique (SMOTE)** [22]. To strictly prevent data leakage, SMOTE was applied exclusively to the training folds after the train/test split. Specifically, the original training set contained 64 short-term and 104 long-term samples. After SMOTE application, the minority class was balanced to achieve 104 samples per class, while the independent test set remained completely uncontaminated.

### XGBoost predictive framework

The core predictive engine used in this study was the **eXtreme Gradient Boosting (XGBoost)** algorithm. The dataset was partitioned into a training set (75%) and a stratified testing set (25%). We configured the XGBoost model with specific hyperparameters to prevent overfitting: learning rate (0.03), tree depth (max_depth=4), and regularization parameters (*gamma* and *subsample*).

### Model interpretability with SHAP analysis

We implemented **SHAP (SHapley Additive exPlanations)** analysis to provide clinically actionable insights. We adopted a bidirectional attribution approach evaluating Global Importance and Directionality (Risk vs. Benefit), allowing for the visualization of both the strength and the nature of the interaction effects.

## Results

### Baseline characteristics

Table 1 summarizes the baseline clinical characteristics of the 210 participants, stratified by their breastfeeding duration. Statistical analysis revealed significant differences in early feeding practices (*P* < 0.001) and milk stasis frequency (*P* = 0.020) between the short-term and long-term groups.

**Table 1:** Baseline Characteristics of the Study Population (*N* = 210).

Variable	Short-term (<11 mo)	Long-term (≥11 mo)	*P*-value
	(*n* = 80)	(*n* = 130)	
**Maternal Age (years)**	29.66 ± 3.40	30.13 ± 3.47	0.337
**Milk Stasis Frequency**	2.52 ± 1.81	3.39 ± 3.53	**0.020**
**Education**, *n* (%)			**0.042**
Low	36 (45.0)	37 (28.5)	
Medium	34 (42.5)	67 (51.5)	
High	10 (12.5)	26 (20.0)	
**Feeding Type**, *n* (%)			**<0.001**
Artificial	5 (6.2)	0 (0.0)	
Mixed	45 (56.2)	41 (31.5)	
Exclusive	30 (37.5)	89 (68.5)	
**Direct Breastfeeding**, *n* (%)			**0.029**
No	13 (16.2)	7 (5.4)	
Partial	17 (21.2)	27 (20.8)	
Yes	50 (62.5)	96 (73.8)	
**Parity**, *n* (%)			**0.044**
Primipara	60 (75.0)	113 (86.9)	
Multipara	20 (25.0)	17 (13.1)	

### Model performance and evaluation

Evaluated on the uncontaminated test set (*N* = 42), the XGBoost model achieved an overall **Accuracy of 76.2%** (95% CI: 64.3%−88.1%), as detailed in Table 2.

**Table 2:** Detailed Performance Metrics of the XGBoost Model.

Class	Precision	Recall	F1-Score	Support
Short Duration (<11 mo)	0.80	0.50	0.62	16
Long Duration (≥11 mo)	0.75	**0.92**	**0.83**	26
**Overall Accuracy**	**0.762**			42
Macro Avg	0.78	0.71	0.72	–
Weighted Avg	0.77	0.76	0.75	–

A critical advantage of our model is its sensitivity to the positive class (Long-term Breastfeeding), achieving an outstanding **Recall of 0.92** and a class-specific **F1-Score of 0.83**. This indicates exceptional effectiveness in identifying mothers with the potential to sustain breastfeeding. The model exhibited a Brier Score of 0.2106, indicating reliable probabilistic calibration for clinical assessment.

**Fig 1:**
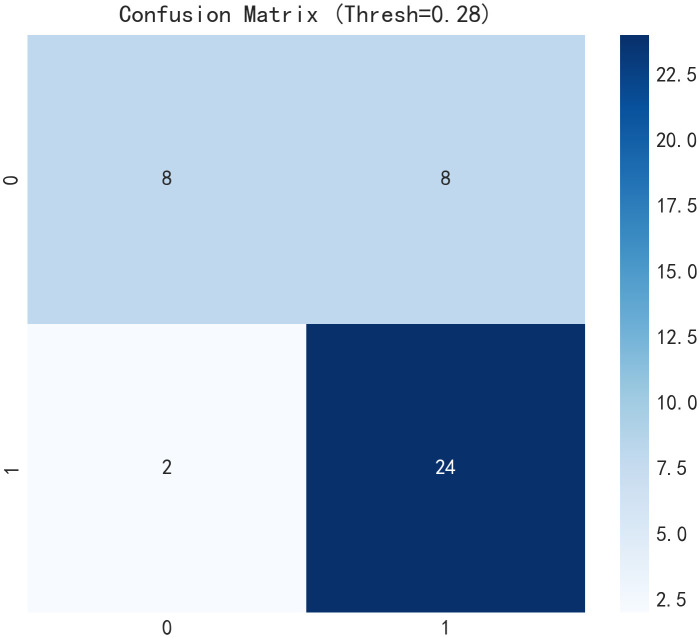
Confusion Matrix. The matrix shows the classification results at the optimal threshold of 0.28.

**Fig 2:**
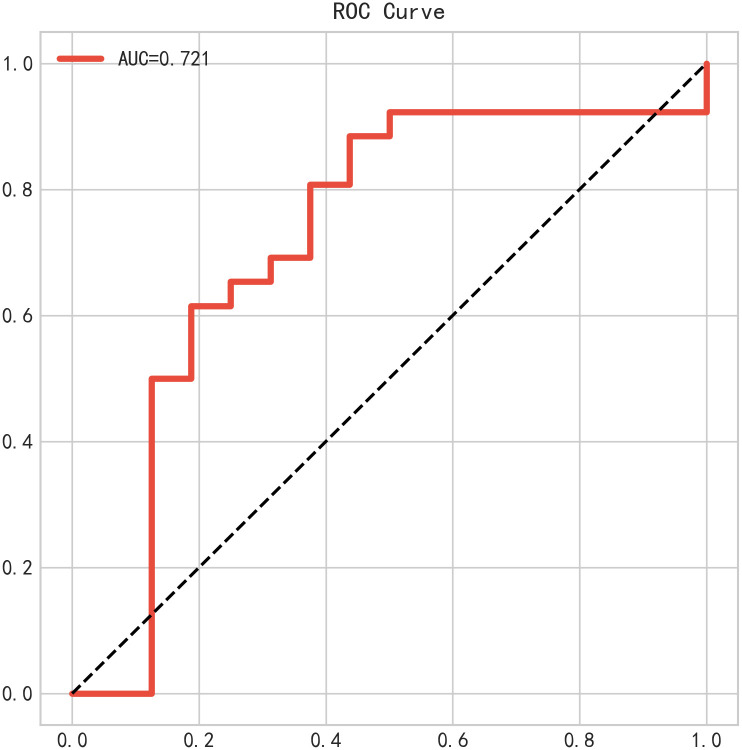
Receiver Operating Characteristic (ROC) Curve. The curve indicates robust discrimination with an AUC of 0.72.

Fig 1 and Fig 2 visualize the discriminative power. The Confusion Matrix (Fig 1) and ROC curve (Fig 2) confirm that the model significantly outperforms random guessing, maintaining a stable balance between sensitivity and specificity.

### Identification of critical interaction features

**Fig 3:**
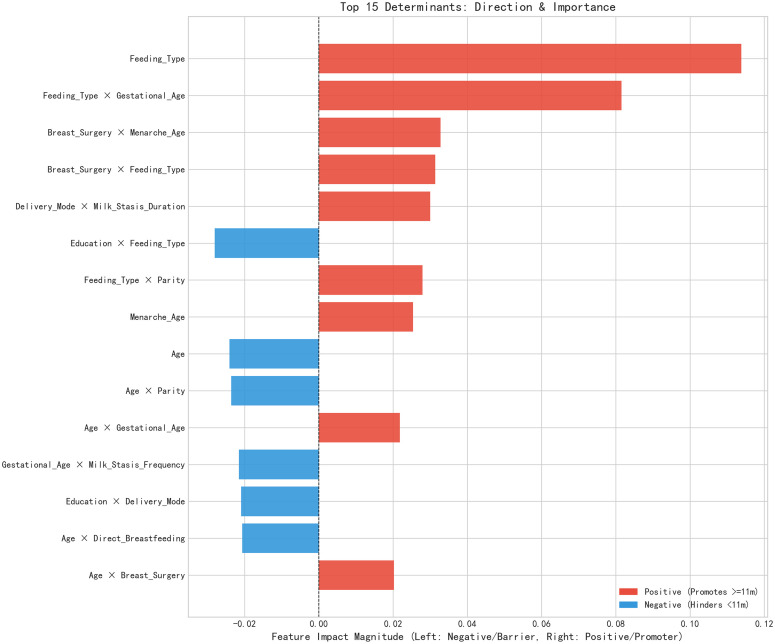
Directional Feature Importance. Horizontal bars represent the feature impact magnitude. Red bars indicate *Positive/Promoting Factors* (positively correlated with duration ≥11 months), while blue bars indicate *Negative/Risk Factors* (correlated with early weaning). Features are ranked by absolute importance.

SHAP analysis was employed to interpret these features. As shown in Fig 3 and Table 3, the model successfully identified non-linear interactions.

**Table 3:** Top 10 Key Predictors and Their Impact Direction based on SHAP Analysis.

Rank	Feature Name	Importance	Impact Direction
1	Feeding_Type	0.1138	Positive (+)
2	Feeding_Type × Gestational_Age	0.0816	Positive (+)
3	Breast_Surgery × Menarche_Age	0.0328	Positive (+)
4	Breast_Surgery × Feeding_Type	0.0313	Positive (+)
5	Delivery_Mode × Milk_Stasis_Duration	0.0300	Positive (+)
6	Education × Feeding_Type	0.0280	Negative (-)
7	Feeding_Type × Parity	0.0280	Positive (+)
8	Menarche_Age	0.0254	Positive (+)
9	Age	0.0240	Negative (-)
10	Age × Parity	0.0236	Negative (-)

Note: “Positive” promotes duration ≥11 months; “Negative” increases risk of < 11 months.

*For Delivery Mode (1 = Vaginal, 0 = Cesarean), a positive interaction means Vaginal delivery protects against stasis risks, implying Cesarean section amplifies the risk.

## Discussion

### Principal findings and interpretation of synergistic effects

This study successfully developed an interpretable XGBoost framework that predicts breastfeeding duration with an accuracy of 76.2% and a high recall of 0.92 for the long-term breastfeeding group. These results align with recent findings [15,16], demonstrating that machine learning algorithms significantly outperform traditional regression.

The analysis revealed a critical interaction between *Delivery Mode* and *Milk Stasis Duration* (Rank 5, Positive correlation). Since vaginal delivery was coded as 1 and Cesarean as 0, this positive correlation signifies that vaginal delivery acts as a protective factor against prolonged milk stasis. Conversely, the negative impact of milk stasis is disproportionately amplified in mothers who underwent Cesarean sections. This reflects a “double burden” mechanism: post-surgical pain and reduced mobility [10] compromise the mother’s physiological resilience to cope with the additional pain of milk stasis.

Furthermore, the negative interaction between *Education Level* and *Feeding Type* (Rank 6) offers a nuanced perspective. While exclusive breastfeeding promotes long-term success, our results indicate that when highly educated mothers transition to mixed feeding, they face a significantly higher risk of early cessation compared to mothers with lower education. We hypothesize that highly educated mothers in China may face greater “return-to-work” pressures and higher opportunity costs [6,7]. Once the strict exclusive breastfeeding routine is broken, they may be more decisive in fully switching to formula feeding as a pragmatic solution. Although our dataset did not directly capture specific employment timelines, this highly plausible mechanism is well-supported by broader sociological literature regarding maternal employment constraints.

### Clinical implications: From general education to precision support

The high recall (0.92) suggests the model is highly effective at identifying mothers with potential for long-term breastfeeding. Clinically, this implies a need for stratified management: Targeted Pain Management for Cesarean mothers to disrupt the “Cesarean-Stasis” negative feedback loop, and Psychosocial Support for highly educated working mothers to address practical solutions for combining work and breastfeeding.

### Methodological advantages

By utilizing SMOTE for class balancing [22] and explicit feature expansion, we overcame small sample limitations [19]. To ensure a fair comparison, all baseline models were trained using the exact same preprocessed feature set (including polynomial expansion and SMOTE balancing) as the proposed XGBoost model. Furthermore, the use of SHAP values provided transparency [21].

### Limitations

Despite the use of SMOTE and cross-validation, the sample size remains limited and derived from a single center. Second, the “Milk Stasis Frequency” was self-reported, potentially introducing recall bias. Future studies should aim for multi-center prospective cohorts.

## Conclusion

Breastfeeding duration is determined by a complex interplay of obstetric, physiological, and social factors. The risk of early weaning is significantly exacerbated by specific combinations of factors, particularly the synergistic negative effects of Cesarean section with milk stasis, and high education with mixed feeding. These findings advocate for a precision medicine approach in lactation support.

## Supporting information

S1 File Supporting information. This file contains the definitions and coding schemes of clinical characteristic variables, performance comparison between XGBoost and baseline models, final hyperparameter settings for the XGBoost model, and the top 10 feature interactions ranked by SHAP importance.
(DOCX)

S1 Data Study dataset. The dataset used for model development and analysis.
(CSV)

## References

[1] Victora CG, Bahl R, Barros AJD, França GVA, Horton S, Krasevec J, et al. Breastfeeding in the 21st century: epidemiology, mechanisms, and lifelong effect. Lancet. 2016;387(10017):475–90. doi: 10.1016/S0140-6736(15)01024-7 26869575

[2] Rollins NC, Bhandari N, Hajeebhoy N, Horton S, Lutter CK, Martines JC, et al. Why invest, and what it will take to improve breastfeeding practices? Lancet. 2016;387(10017):491–504. 26869576 10.1016/S0140-6736(15)01044-2

[3] Bhattacharjee NV, Schaeffer LE, Hay SI, Lu D, Schipp MF, Local Burden of Disease Exclusive Breastfeeding Collaborators. Mapping Inequalities in Exclusive Breastfeeding in Low- and Middle-Income Countries, 2000–2018. Nat Hum Behav. 2021;5(8):1027–45. 34083753 10.1038/s41562-021-01108-6 PMC8373614

[4] Zong X, Wu H, Zhao M, Magnussen CG, Xi B. Global Prevalence of WHO Infant Feeding Practices in 57 LMICs in 2010–2018 and Time Trends since 2000 for 44 LMICs. eClinicalMedicine. 2021;37:100971. 34386748 10.1016/j.eclinm.2021.100971 PMC8343261

[5] Li Q, Tian J, Xu F, Binns C. Breastfeeding in China: A Review of Changes in the Past Decade. Int J Environ Res Public Health. 2020;17(21):8234. doi: 10.3390/ijerph17218234 33171798 PMC7664678

[6] Yang Z, Ding Y, Song S, Zhang Y, Li A, Su M, et al. Factors Affecting the Breastfeeding Duration of Infants and Young Children in China: A Cross-Sectional Study. Nutrients. 2023;15(6):1353. doi: 10.3390/nu15061353 36986082 PMC10051738

[7] Duan Y, Yang Z, Bi Y, Wang J, Pang X, Jiang S, et al. What are the determinants of low exclusive breastfeeding prevalence in China? A cross-sectional study. Matern Child Nutr. 2022;18(2):e13324. doi: 10.1111/mcn.13324 35137523 PMC8932722

[8] Cohen SS, Alexander DD, Krebs NF, Young BE, Cabana MD, Erdmann P, et al. Factors Associated with Breastfeeding Initiation and Continuation: A Meta-Analysis. J Pediatr. 2018;203:190-196.e21. doi: 10.1016/j.jpeds.2018.08.008 30293638

[9] Valdés V, Schooley J. The Role of Education in Breastfeeding Success. Food Nutr Bull. 1996;17(4):1–7. doi: 10.1177/156482659601700427

[10] Hobbs AJ, Mannion CA, McDonald SW, Brockway M, Tough SC. The impact of caesarean section on breastfeeding initiation, duration and difficulties in the first four months postpartum. BMC Pregnancy Childbirth. 2016;16:90. doi: 10.1186/s12884-016-0876-1 27118118 PMC4847344

[11] Prior E, Santhakumaran S, Gale C, Philipps LH, Modi N, Hyde MJ. Breastfeeding after cesarean delivery: a systematic review and meta-analysis of world literature. Am J Clin Nutr. 2012;95(5):1113–35. 22456657 10.3945/ajcn.111.030254

[12] Gianni ML, Bettinelli ME, Manfra P, Sorrentino G, Bezze E, Plevani L. Breastfeeding Difficulties and Risk for Early Breastfeeding Cessation. Nutrients. 2019;11(10):2266. 31547061 10.3390/nu11102266 PMC6835226

[13] Kvist LJ, Larsson BW, Hall-Lord ML, Steen A, Schalén C. The role of bacteria in lactational mastitis and some considerations of the use of antibiotic treatment. Int Breastfeed J. 2008;3:6. doi: 10.1186/1746-4358-3-6 18394188 PMC2322959

[14] Lathouwer SD, Lionet C, Lansac J, Body G, Perrotin F. Predictive factors of early cessation of breastfeeding. Eur J Obstet Gynecol Reprod Biol. 2004;117(2):169–73. doi: 10.1016/j.ejogrb.2003.10.041 15541852

[15] Nejsum FM, Wiingreen R, Jensen AK, Løkkegaard ECL, Mølholm Hansen B. Predicting early cessation of exclusive breastfeeding using machine learning techniques. PLoS One. 2025;20(1):e0312238. doi: 10.1371/journal.pone.0312238 39787191 PMC11717195

[16] Yusuf JO, Miyim AM. Predicting breastfeeding practice of Nigerian child using machine learning and deep learning algorithms. dujopas. 2022;7(4a):183–93. doi: 10.4314/dujopas.v7i4a.19

[17] Rajkomar A, Dean J, Kohane I. Machine Learning in Medicine. N Engl J Med. 2019;380(14):1347–58. doi: 10.1056/NEJMra1814259 30943338

[18] Chen T, Guestrin C. XGBoost: A Scalable Tree Boosting System. In: Proceedings of the 22nd ACM SIGKDD International Conference on Knowledge Discovery and Data Mining. ACM; 2016. p. 785–94.

[19] Shaikhina T, Khovanova NA. Handling limited datasets with neural networks in medical applications: A small-data approach. Artif Intell Med. 2017;75:51–63. doi: 10.1016/j.artmed.2016.12.003 28363456

[20] Adadi A, Berrada M. Peeking Inside the Black-Box: A Survey on Explainable Artificial Intelligence (XAI). IEEE Access. 2018;6:52138–60. doi: 10.1109/access.2018.2870052

[21] Markus AF, Kors JA, Rijnbeek PR. The role of explainability in creating trustworthy artificial intelligence for health care: A comprehensive survey of the terminology, design choices, and evaluation strategies. J Biomed Inform. 2021;113:103655. doi: 10.1016/j.jbi.2020.103655 33309898

[22] Chawla NV, Bowyer KW, Hall LO, Kegelmeyer WP. SMOTE: Synthetic Minority Over-sampling Technique. jair. 2002;16:321–57. doi: 10.1613/jair.953

