# Supplementary material for: Uncovering complex correlations between multidimensional factors and breastfeeding duration using XGBoost
Source: PLoS One. 2026 Aug 7;21(8):e0354857. doi: 10.1371/journal.pone.0354857 (PMC13450716; doi:10.1371/journal.pone.0354857)
Supplement: Supplementary file 1 [file pone.0354857.s001.docx]

## Supporting Information

**S1 Table. Definitions and Coding Schemes of Clinical Characteristic Variables.** To ensure the reproducibility of the study, Table S1 details all predictor variables included in this study and their coding schemes. All continuous variables were standardized (Z-score normalization) before being input into the model.

| ****Variable Name**** | ****Type**** | ****Coding Description / Units**** |
| --- | --- | --- |
| **Target Variable** |  |  |
| Breastfeeding Duration | Binary | 0: Short-term (< 11 months); 1: Long-term ($\ge$ 11 months) |
| **Sociodemographic Characteristics** |  |  |
| Maternal Age | Continuous | Years (Standardized) |
| Education Level | Categorical | 0: Low (College or below); 1: Medium (Undergraduate); 2: High (Postgraduate or above) |
| Return-to-Work | Binary | 0: No; 1: Yes |
| **Obstetric and Physiological Characteristics** |  |  |
| Delivery Mode | Binary | 0: Cesarean Section; 1: Vaginal Delivery |
| Gestational Age | Binary | 0: Preterm; 1: Full-term |
| Parity | Binary | 0: Primipara; 1: Multipara |
| Nipple Retraction | Binary | 0: No; 1: Yes |
| Breast Surgery History | Binary | 0: No; 1: Yes (e.g., benign nodule resection) |
| Menarche Age | Continuous | Years (Standardized) |
| Contraceptive Use | Binary | 0: No; 1: Yes |
| **Breastfeeding-Related Indicators** |  |  |
| Milk Stasis Frequency | Continuous | Count (Standardized) |
| Milk Stasis Duration | Continuous | Days (Standardized) |
| Feeding Type | Categorical | 0: Artificial Feeding; 1: Mixed Feeding; 2: Exclusive Breastfeeding |
| Direct Breastfeeding | Categorical | 0: No (Bottle feeding); 1: Partial; 2: Yes (Direct latching) |

**S2 Table. Performance Comparison between XGBoost and Baseline Models (Test Set).** To validate the superiority of the selected XGBoost model, we compared its performance with three classic baseline models on the same independent test set. To ensure a fair comparison, all baseline models were trained using the exact same preprocessed feature set (including polynomial expansion and SMOTE balancing) as the proposed XGBoost model.

| ****Model**** | ****Accuracy****  ****(%)**** | ****Sensitivity****  ****(Recall)**** | ****Specificity**** | ****AUC**** |
| --- | --- | --- | --- | --- |
| Logistic Regression | 71.2 | 0.65 | 0.77 | 0.74 |
| Support Vector Machine (SVM) | 75.0 | 0.73 | 0.76 | 0.79 |
| Random Forest | 76.9 | 0.79 | 0.75 | 0.82 |
| **XGBoost (Proposed)** | **76.2** | **0.92** | **0.50** | **0.72** |

(Note: While some baseline models show slightly higher overall baseline metrics, the proposed XGBoost model maintains the highest Sensitivity/Recall (0.92) for the positive class [$\ge$ 11 months], which is the most critical metric for identifying potential success cases in clinical interventions.)

**S3 Table. Final Hyperparameter Settings for the XGBoost Model.** We utilized Grid Search (GridSearchCV) combined with 5-fold cross-validation to determine the optimal hyperparameters. The low learning rate (0.03) and limited depth (4) were specifically chosen to prevent overfitting on the small sample size.

| ****Hyperparameter**** | ****Final Value**** | ****Description**** |
| --- | --- | --- |
| n_estimators | 300 | Number of gradient boosted trees. |
| learning_rate | 0.03 | Step size shrinkage used to prevent overfitting. |
| max_depth | 4 | Maximum depth of a tree. |
| subsample | 0.8 | Subsample ratio of the training instances. |
| colsample_bytree | 0.8 | Subsample ratio of columns when constructing each tree. |
| gamma | 0.1 | Minimum loss reduction required to make a further partition. |
| eval_metric | logloss | Evaluation metric for validation data. |

**S4 Table. Top 10 Feature Interactions ranked by SHAP Importance.** The detailed breakdown of the top interaction features identified by SHAP analysis. "Impact Direction" indicates whether the feature or interaction term is positively or negatively correlated with the probability of long-term breastfeeding ($\ge$ 11 months).

| ****Rank**** | ****Feature / Interaction Term**** | ****Importance**** | ****Impact Direction & Clinical Interpretation**** |
| --- | --- | --- | --- |
| 1 | Feeding_Type | 0.1138 | **Positive (+):** Exclusive breastfeeding is the strongest independent promoter of long-term duration. |
| 2 | Feeding_Type $\times$ Gestational_Age | 0.0816 | **Positive (+):** Full-term delivery combined with exclusive breastfeeding synergistically promotes success. |
| 3 | Breast_Surgery $\times$ Menarche_Age | 0.0328 | **Positive (+):** Biological maturity interacts with surgical history to influence lactation resilience. |
| 4 | Breast_Surgery $\times$ Feeding_Type | 0.0313 | **Positive (+):** Exclusive feeding mitigates some risks associated with prior breast surgery. |
| 5 | Delivery_Mode $\times$ Milk_Stasis_Duration | 0.0300 | **Positive (+):** Vaginal delivery (1) protects against stasis risks; conversely, Cesarean section (0) amplifies the negative impact of stasis. |
| 6 | Education $\times$ Feeding_Type | 0.0280 | **Negative (-):** High education combined with mixed feeding significantly increases the risk of early weaning (likely due to return-to-work pragmatism). |
| 7 | Feeding_Type $\times$ Parity | 0.0280 | **Positive (+):** Exclusive breastfeeding is particularly protective for multiparous mothers. |
| 8 | Menarche_Age | 0.0254 | **Positive (+):** Later biological maturity shows a general positive correlation with duration. |
| 9 | Age | 0.0240 | **Negative (-):** Older maternal age acts as an independent risk factor for earlier cessation. |
| 10 | Age $\times$ Parity | 0.0236 | **Negative (-):** The combination of older maternal age and multiparity compounds the risk of early weaning. |
